# Objective measurement of tinnitus using functional near-infrared spectroscopy and machine learning

**DOI:** 10.1371/journal.pone.0241695

**Published:** 2020-11-18

**Authors:** Mehrnaz Shoushtarian, Roohallah Alizadehsani, Abbas Khosravi, Nicola Acevedo, Colette M. McKay, Saeid Nahavandi, James B. Fallon

**Affiliations:** 1 The Bionics Institute, East Melbourne, Victoria, Australia; 2 Medical Bionics Department, The University of Melbourne, Melbourne, Australia; 3 Institute for Intelligent Systems Research and Innovation (IISRI), Deakin University, Melbourne, Australia; 4 Department of Otolaryngology, The University of Melbourne, Melbourne, Australia; Politecnico di Milano, ITALY

## Abstract

Chronic tinnitus is a debilitating condition which affects 10–20% of adults and can severely impact their quality of life. Currently there is no objective measure of tinnitus that can be used clinically. Clinical assessment of the condition uses subjective feedback from individuals which is not always reliable. We investigated the sensitivity of functional near-infrared spectroscopy (fNIRS) to differentiate individuals with and without tinnitus and to identify fNIRS features associated with subjective ratings of tinnitus severity. We recorded fNIRS signals in the resting state and in response to auditory or visual stimuli from 25 individuals with chronic tinnitus and 21 controls matched for age and hearing loss. Severity of tinnitus was rated using the Tinnitus Handicap Inventory and subjective ratings of tinnitus loudness and annoyance were measured on a visual analogue scale. Following statistical group comparisons, machine learning methods including feature extraction and classification were applied to the fNIRS features to classify patients with tinnitus and controls and differentiate tinnitus at different severity levels. Resting state measures of connectivity between temporal regions and frontal and occipital regions were significantly higher in patients with tinnitus compared to controls. In the tinnitus group, temporal-occipital connectivity showed a significant increase with subject ratings of loudness. Also in this group, both visual and auditory evoked responses were significantly reduced in the visual and auditory regions of interest respectively. Naïve Bayes classifiers were able to classify patients with tinnitus from controls with an accuracy of 78.3%. An accuracy of 87.32% was achieved using Neural Networks to differentiate patients with slight/ mild versus moderate/ severe tinnitus. Our findings show the feasibility of using fNIRS and machine learning to develop an objective measure of tinnitus. Such a measure would greatly benefit clinicians and patients by providing a tool to objectively assess new treatments and patients’ treatment progress.

## Introduction

Tinnitus is a condition characterised by hearing unwanted sounds that are not present externally. Chronic tinnitus affects around 6–20% of adults with approximately 20% of these experiencing it in a severe form with symptoms such as depression, cognitive dysfunction and stress [1, 2]. Despite its wide prevalence, there is currently no clinically used objective test that can measure changes in brain activity related to tinnitus. There is no objective way to determine the presence or severity of tinnitus, or assess whether treatments are effective [3]. Developing an objective measure of tinnitus, the primary aim of this study, will allow more accurate assessment of this condition and is an important step in developing and measuring the effectiveness of potential treatments currently being developed.

Both experimental animal and human studies have shown changes in the central nervous system associated with tinnitus [1, 2, 4, 5]. These changes have been identified at a variety of locations along the auditory pathway, in the auditory cortex and more recently in non-auditory brain areas [1, 2, 6]. A review by Elgoyhen et al. proposed that tinnitus results from abnormal activity in multiple overlapping brain networks with variation in involvement of each of these networks leading to the diverse manifestation of this condition [7]. Activation of the auditory cortex in the resting state has been proposed to reflect the loudness of the tinnitus [7]. Co-activation of non-auditory regions such as frontal areas and the limbic system have been associated with aspects of tinnitus such as distress [8]. Neural changes shown to be associated with tinnitus include changes in the level of spontaneous neural activity, changes in neural synchrony and reorganisation of cortical tonotopic maps [2].

Different functional imaging techniques have been used to localise brain areas related to tinnitus [2, 9]. The most common methods are electroencephalography (EEG) [10], magnetoencephalography (MEG) [11, 12], positron emission tomography (PET) [13] and functional magnetic resonance imaging (fMRI) [4]. In this study we used functional near-infrared spectroscopy (fNIRS) to record resting state and evoked responses, two widely used paradigms used in functional neuroimaging of tinnitus [2]. fNIRS has great potential to transform clinical practice as it is non-invasive and non-radioactive (unlike PET) and, importantly for routine clinical use, is quiet, portable and cost-effective. Similar to fMRI, fNIRS measures changes in blood oxygen levels in the brain; however, fNIRS has better temporal resolution and does not produce scanner noise making it more suited to hearing related research. A limitation of fNIRS is its inability to image deep cortical regions; however, in the research described below, we have focused on cortical regions accessible using fNIRS which have been associated with tinnitus using other imaging techniques.

A number of cortical regions identified in tinnitus neuroimaging studies, include the auditory cortex, frontal cortex and cuneus regions [9, 14, 15]. In particular, increased resting state activity in frontal regions has been attributed to tinnitus distress, salience or attentional focus [9]. A meta-analysis of nine studies comparing resting state brain activity in patients with tinnitus compared to controls, found reduced activity in the cuneus in tinnitus patients [9]. The cuneus is located in the occipital part of the brain and is involved in visual processing. It has been proposed that, due to the existence of neural pathways between auditory and visual regions, tinnitus-related abnormal activity in the auditory cortex can lead to altered activity in the cuneus [9, 16]. The subjective loudness of tinnitus has been proposed to be associated with integration of multi-sensory, i.e. audio-visual, information [17].

The cortical regions discussed above are accessible using fNIRS as shown in previous studies [18–20]. Our previous research has shown feasibility of recording auditory responses using fNIRS and has demonstrated responses to be dependent on the intensity of auditory stimuli [18]. The auditory region is positioned relatively deep in the cortex and previous work suggests that recorded signals in adults do not originate from the primary auditory cortex and instead are more likely to be from the auditory para-belt regions [21].

A number of studies have recently used fNIRS in participants with tinnitus to record brain activity at rest or in response to sound, showing sensitivity of fNIRS to detect tinnitus-related neural activity [5, 22–24]. Resting state data can be used to derive measures of connectivity between different regions of the brain. It has been reported that there are differences in brain connectivity between participants with tinnitus and those without [22]; however, the two groups in that study were not age-matched. Promisingly, another study reported changes in fNIRS auditory responses in participants with tinnitus as a result of applying transcranial magnetic stimulation, a potential tinnitus therapy [23].

Most reported findings in brain imaging studies on tinnitus are based on statistical analysis of either resting state or evoked brain activity. Machine learning algorithms are used to find patterns in complex data with non-linear relationships as is the case with brain imaging signals [25]. Machine learning methods are well suited to rich datasets such as fNIRS features from multiple channels, different conditions (i.e. resting state and evoked) and demographic data. These methods are also suited for development of clinically usable measures as they enable a reduction of the number of fNIRS channels or source-detector pairs required as the information in some may be contained in others or channels might be identified that have no information associated with tinnitus. Subjective ratings of tinnitus severity can be used with machine learning techniques to map fNIRS signal features to severity levels (*training* phase). fNIRS features from other individuals can then be classified to tinnitus severity levels based on past observations. This method has been successfully used to classify similar conditions such as pain type with an accuracy of 90% using 13 fNIRS features [26].

In this study, we aimed to apply statistical and machine learning algorithms to fNIRS signals to: 1) assess the sensitivity of fNIRS to differentiate individuals with tinnitus from controls, and 2) identify fNIRS features associated with subjective ratings of tinnitus severity and whether these could differentiate between perceived loudness of tinnitus and annoyance. To avoid machine learning models becoming a ‘black box’ with little information about features and parameters, we have first performed statistical analysis to gain a better understanding of signal features, cortical regions and conditions that show group differences and changes with tinnitus severity levels. This ensured that our machine learning models provided physiologically relevant information. A reliable objective measure of tinnitus will enable monitoring of changes as a result of potential treatments. The clinical management of patients with tinnitus would greatly benefit from such a tool.

## Methods

### Participants

**Fig 1:**
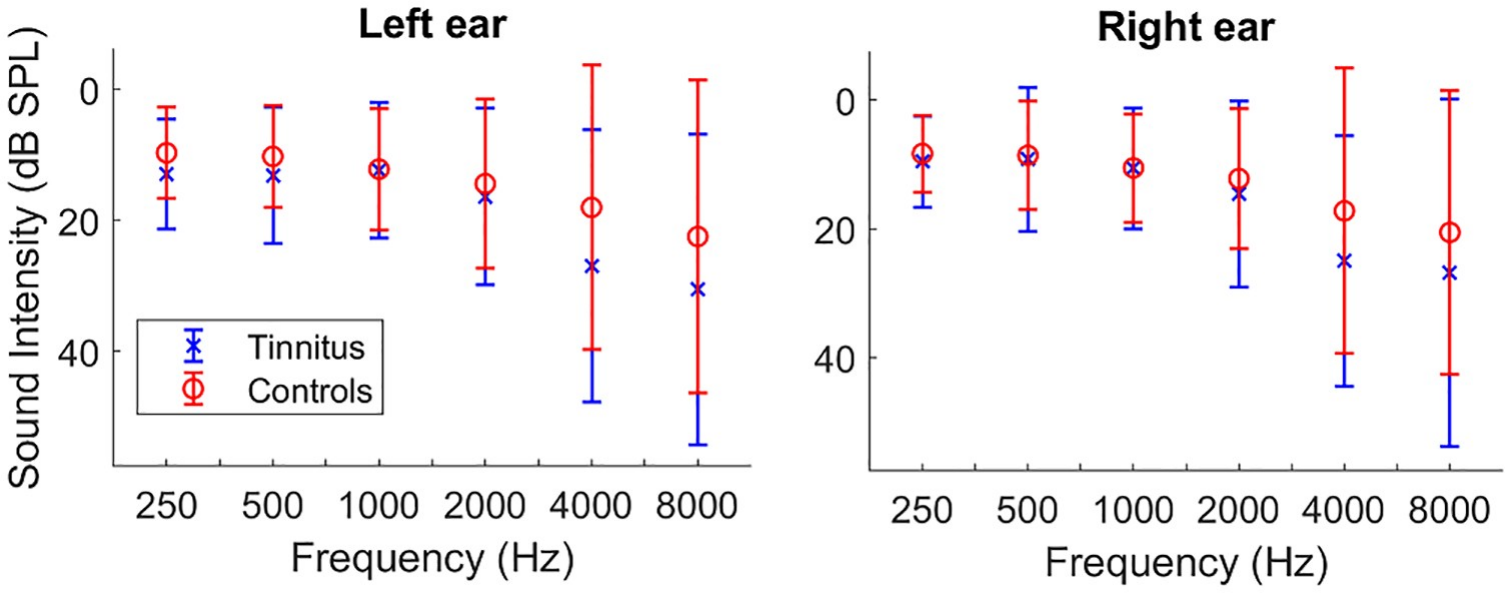
Mean (SD) of hearing thresholds in participants with tinnitus and controls.

The study was approved by the Royal Victorian Eye and Ear Hospital Human Research Ethics Committee (project number 17/1332H). Written informed consent was obtained from all participants. Twenty five participants with chronic subjective tinnitus (23 experiencing it bilaterally) were recruited via advertisement through local audiology clinics and Bionics Institute social media. Twenty-one healthy adults with no history of tinnitus, neurological or hearing disorders were also tested. Data from three healthy participants were excluded, two due to long hair and poor signal quality and one due to technical issues with the cap. Participants attended one testing session. Pure tone audiometry was performed on all participants at frequencies of 0.25, 0.5, 1, 2, 4, and 8 kHz (Fig 1). For the participants included in the study, hearing thresholds averaged across frequencies for each ear were not significantly different between groups (left ear: *t*(41) = −1.13, *p* = 0.26; right ear: *t*(41) = −0.81, *p* = 0.42). Hearing thresholds at 4 and 8 KHz were also compared between groups as these showed higher loss in the tinnitus group (Fig 1). No significant group difference was found at 4 (left: *t*(41) = −1.37, *p* = 0.18; right: *t*(41) = −1.22, *p* = 0.23)) and 8 KHz (left: *t*(41) = −1.1, *p* = 0.28; right: *t*(41) = −0.81, *p* = 0.42)). There was no significant difference in mean age between the two groups (t(41) = -0.65, *p* = 0.51). Tinnitus severity was assessed using the Tinnitus Handicap Inventory (THI) [27]. The THI is a 25-item test which quantifies the perceived severity of tinnitus on a scale of 0–100. Score ranges are associated with different severity levels (e.g. 0–16 slight tinnitus, 58–76 severe). Participants with tinnitus were also asked to rate the loudness and annoyance of their tinnitus on a scale of 1 to 10 before each recording. Demographic and clinical data are shown in Table 1.

**Table 1:** Participant demographics.

	Controls	Tinnitus
No. of participants	18	25
gender (male: female)	11:7	16:9
Age, mean (SD), range	45.5 (16.7), 25–76	48.4 (12.9), 25–68
Handedness	R: 18	R: 21, L: 2, both: 2
THI, mean (SD), range	N/A	26.2 (17.1), 4–60
Tinnitus duration, mean (SD), range	N/A	11.5 (8.8), 0.5–25
Tinnitus laterality	N/A	R: 2, bilateral: 23

THI, Tinnitus Handicap Inventory; R, right; L, left; Tinnitus duration: length of time patients have experienced tinnitus.

### fNIRS recordings

**Fig 2:**
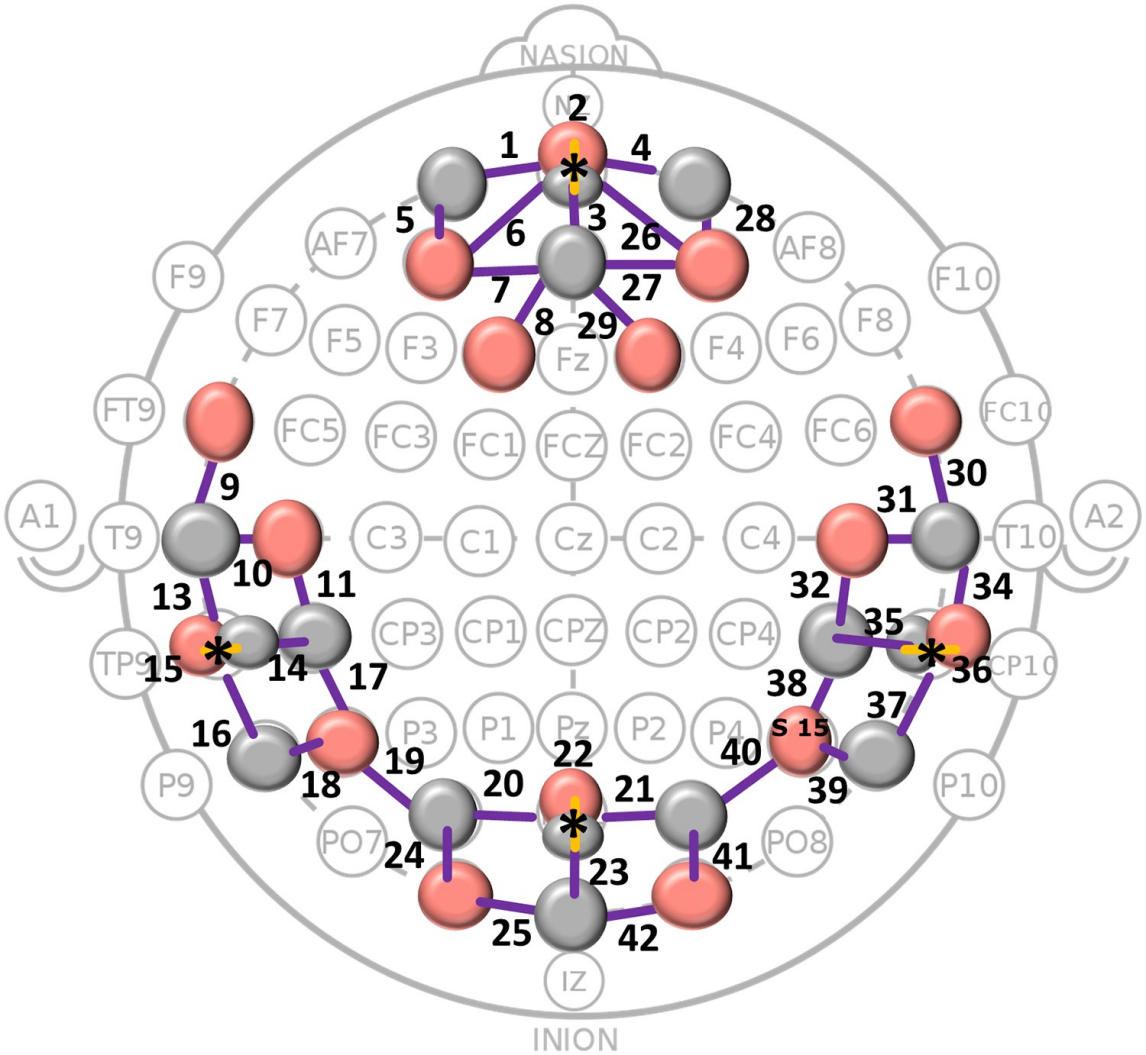
fNIRS montage. Sixteen sources (red circles) and 16 detectors (grey circles) forming *channels* were placed on frontal, temporal and occipital regions of the scalp. Channel numbers are shown. 36 long and 4 short channels (marked by * and yellow source-detector links) were formed.

A multi-channel continuous-wave fNIRS system operating at 760 and 850 nm (NIRScout, NIRx Medical Technologies LLC) was used to collect data. A total of 16 sources and 16 detectors were placed over the frontal, temporal and occipital cortical regions (Fig 2). Each source-detector pair forms a *channel*. Sources and detectors were arranged using NIRSite software (NIRx Medical Technologies LLC) which uses the ICBM-152 head model and allows exporting MNI coordinates corresponding to channel locations. These coordinates were then used in AtlasViewer software [28] to determine the brain region corresponding to each channel location and to ensure the auditory and visual cortex in particular (due to the use of auditory and visual stimuli) were covered. Channels over or around the auditory cortex were sited to achieve good fNIRS response signals to auditory stimuli based on our previous work which showed fNIRS responses to auditory stimuli at different intensities [18]. Source-detector pairs were placed 30mm apart, forming 36 *long* channels. In each of the four cortical regions, a ‘short’ channel was formed by placing a source-detector pair 11mm apart. fNIRS signals contain systemic signals from superficial layers of the head including the scalp and skull, which interfere with detecting deeper cortical signals. Using source-detector pairs which are placed closer together, systemic signals from superficial layers can be recorded. These can then be used to remove the systemic artefacts from the long channels as described below.

### Auditory stimulation

Auditory stimuli were delivered binaurally via audiometric insert earphones (ER-3A insert earphone, E-A-RTONE^™^ 165 GOLD, USA). Stimuli consisted of 15-second segments of pink noise calibrated using a Norsonic sound level meter (Norsonic SA, Norway) and delivered at 65 dB Sound Pressure Level (SPL). The power in pink noise is inversely proportional to the signal frequency with equal power in different octaves (i.e. doubling of frequencies). This is similar to how the human auditory system perceives sound.

### Visual stimulation

The visual stimulus was a reversing display of circular checkerboard patterns with pattern reversal at a temporal frequency of 7.5 Hz (15 reversals per second). This pattern was used as it produces strong cortical responses in people with good visual acuity [19]. The images were radial in nature and consisted of rings, divided into sectors with neighbouring sectors of opposite colour (black and white).

### Experimental design

fNIRS testing was performed in a sound-treated booth. Participants sat on a comfortable chair and auditory and visual stimuli were presented using Presentation software (Neurobehavioral Systems, USA).

**Fig 3:**
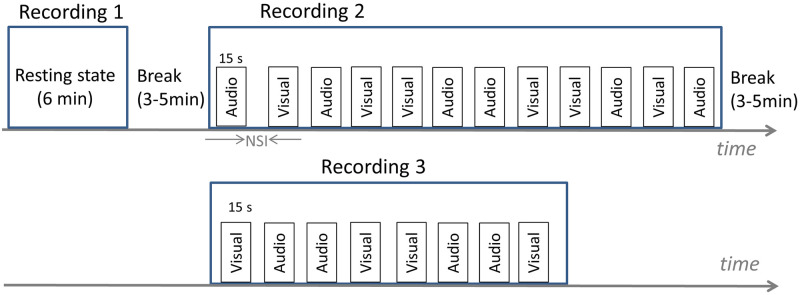
fNIRS experiment block design. Three recordings were performed during each testing session with 3–5 minute rest breaks in-between. In ‘recording period 2’, six 15-second auditory and six 15- second visual stimuli were applied. A 3–5 minute break was then given to participants and then ‘recording period 3’ was performed to collect a further four auditory and four visual trials. All together, 10 trials of each stimulus type were collected. Stimuli were randomised across blocks with no more than two stimuli of the same type in a row. Non-stimulus intervals between stimuli within a recording period were 20 or 25 seconds long. NSI: non-stimulus interval.

The fNIRS test session consisted of three recording periods with short rest breaks given in between (Fig 3). The first recording period was a six-minute resting-state recording. During this recording period, participants were instructed to sit still with their eyes closed but not fall asleep. The second and third recording periods were composed of auditory or visual stimulus blocks (Fig 3). Stimuli were randomised across blocks with no more than two blocks of the same type in a row and 20 or 25 second non-stimulus intervals between stimuli. In total, each stimulus type was repeated 10 times (six in recording period 2 and four in recording period 3). The total recording time (excluding short rest breaks) was approximately 20 minutes. Data was recorded at a sampling rate of 7.8125 Hz per channel.

### Analysis

Data processing was performed in Matlab 2019a (Mathworks, USA). Pre-processing of fNIRS signals was performed using NIRS Brain AnalyzIR Toolbox [29] and custom written Matlab scripts. Channels with poor signal quality were identified using the following criteria and excluded from further analysis. First, channels with gains over 7 showing inadequate detected light intensity were rejected. The gain is calculated by the NIRx device during a calibration procedure performed prior to each experiment. In the NIRx system, gain values below 7 are defined as optical signals within the range 0.09–1.4 V and noise levels less than 2.5% (NIRstart 14.2 User Manual). Channels were also checked for their cardiac signal content as this is a sign of good contact between optodes and the scalp [30, 31]. This was done using a scalp coupling index (SCI) which is calculated by band-pass filtering the two detected signals at 760 and 850 nm between 0.2 to 2.5 Hz [30]. Signals from optodes with good skin contact will mainly contain heart rate data and hence be highly correlated. Channels with SCI values less than 0.75 were rejected. On average 13% of channels were rejected.

For the remaining channels the following processing was applied. For resting state recordings, the original unfiltered signals from each channel were down-sampled to 1Hz and converted to optical density [32]. For evoked response recordings, conversion to optical density was performed at the original sampling rate. Short channel correction was applied to optical density data using the function *ntbxSSR*.*m* in the NIRS toolbox (parameter *task* set to 0) [33, 34] to remove extracerebral signals from long channels. The corrected optical density in each long channel was calculated by subtracting a fraction of the closest short channel [33]. This subtraction removes two sources of interference, fluctuations measured from the scalp and global fluctuations such as systemic responses and respiration. Concentration changes of oxygenated and de-oxygenated haemoglobin (HbO and HbR respectively) were then estimated using the modified Beer-Lambert law [35].

### Resting state

Seed analysis is commonly used to investigate resting-state functional connectivity networks in the brain [4, 5]. In this method, a cortical region is selected as the *seed* and its connectivity with other regions is examined by finding correlations between the seed region and other brain regions (e.g. see [5]). In this study, two channels over the temporal cortex were chosen on each side of the head. Channels 9 and 10 on the left side and 30 and 31 on the right, were estimated to cover the superior temporal and Heschl’s gyrus (Table 2). Signals from the two channels on each side were then averaged and used as a seed. Correlations between seed channels and the other channels were calculated using *whitened correlations* (NIRS toolbox function *nirs*.*sFC*.*ar_corr*.*m*) [32]. This robust correlation method addresses the sensitivity of fNIRS to false correlations due to the slow hemodynamic signal, systemic physiological noise such as heart rate and breathing (serial correlations) and motion artefacts which can introduce non-normal noise structures. Values obtained for channels comprising frontal and occipital regions of interest (ROI) were then averaged for statistical analysis. The frontal ROI included channels over the superior frontal gyrus, medial, superior frontal gyrus, medial orbital and middle frontal gyrus (channels 1, 3, 4, 5, 6, 7, 8, 26, 27, 28, 29). The occipital ROI chosen covered the cuneus and superior occipital gyrus (channels 20, 21, 23, 24, 25, 41, 42). Whitened correlations were derived from both HbO and HbR signals and compared between groups.

**Table 2:** Anatomical region associated with each channel number.

Left side		Right side	
Channel no.	Cortical region	Channel no.	Cortical region
9	Superior temporal gyrus	30	Superior temporal gyrus
10	Superior temporal gyrus	31	Heschl’s gyrus
11	Supramarginal gyrus	32	Supramarginal gyrus
13	Middle temporal gyrus	34	Middle temporal gyrus
14	Middle temporal gyrus	35	Superior temporal gyrus
16	Inferior temporal gyrus	37	Middle temporal gyrus
17	Angular gyrus	38	Angular gyrus
18	Middle temporal gyrus	39	Middle temporal gyrus

### Evoked responses

To analyse evoked responses, motion artefacts were removed using the function *WaveletFilter* (outlier threshold *s*et to 3). Signals were band-pass filtered between 0.01–0.12 Hz by applying zero-phase 8^th^ order Butterworth high-pass (at 0.01 Hz) and low-pass (0.12 Hz) filters respectively. HbO and HbR concentrations were then estimated using the modified Beer-Lambert law. For each channel, HbO and HbR signals were epoched from t = -5 to t = 30 relative to stimulus onset using the *EpochExtraction* function which removes linear trends and baseline corrects epochs by subtracting the baseline mean. Based on an outlier detection function [36] epochs with amplitudes exceeding 2.5 standard deviations above the epoch mean were rejected. For each of the conditions recording auditory and visual responses, mean HbO and HbR activation across time windows 0 to 5 seconds (for auditory responses) and 10–15 seconds (visual) were calculated. These time windows were chosen to capture the initial rising phase of the response based on grand averaged group responses and waveform morphology (further described in the results section). fNIRS features from evoked responses were averaged over ROIs for statistical analysis. Left and right temporal ROIs included channels listed in Table 2 along with the estimated anatomical regions these channels covered. Visual evoked responses were averaged over the occipital ROI mentioned for resting data, with channels covering the cuneus and superior occipital gyrus (channels 20, 21, 23, 24, 25, 41, 42).

### Statistical analysis

Data normality was tested using residual normal probability plots. For resting state and visual response measures, group comparisons were performed using Independent sample t-tests. Auditory responses were compared among regions (left and right temporal) using paired t-tests and among groups using independent sample t-tests. Multiple linear regression was used to investigate relationships between fNIRS features which showed significant between-group differences, with behavioural measures and demographics.

Statistical analyses were performed using IBM SPSS Statistics for Windows, V26 (IBM Corp.). A value of p < 0.05 was considered statistically significant.

### Machine learning

To combine features from resting state and evoked response signals from fNIRS channels over different cortical regions, machine learning methods including feature selection and classifiers were used. Features input to these algorithms included auditory and visual response amplitudes and frontal and occipital connectivity measures described above. Here, features from all channels were used as individual inputs (and not averaged over ROIs) to allow the feature selection algorithms to automatically select channels which can best distinguish between groups. Both HbO and HbR- derived features were used. Information Gain was used to select the most relevant features by ranking them based on their weight or importance in classification. Information Gain is a measure of entropy in the data and enables identification of channels and HbO/ HbR features with the most relevant information for classification. These features were then used with four different classification methods to classify participants as controls or experiencing tinnitus. Classifiers were also used to differentiate the patients with tinnitus as having slight/ mild versus moderate/ severe tinnitus (based on THI ratings). In the latter analysis data was categorised into two groups only, to increase the sample size in each. The four classifiers used were Naïve Bayes, K-nearest neighbor (KNN), Rule Induction and Artificial neural networks (ANN). These established algorithms have been previously used for similar applications such as medical diagnosis and classification of types of human pain [26, 37].

Connectivity measures and evoked response amplitudes described above and derived from both HbO and HbR were used with classifiers. Classifier performance was assessed using only connectivity measures, only evoked response features or using both connectivity and evoked features to assess the relative importance of the different features. To calculate the performance of these algorithms, 10-fold cross validation was used. This validation method randomly partitions the dataset into 10 subsets. One subset is kept for testing while the other nine are used for training. This process is iterated throughout the whole 10 subsets (each time using one of the 10 subsets for testing) and the average sensitivity (true positive rate), specificity (true negative rate) and accuracy of the classifier is calculated. Classification accuracy or predictive performance was calculated as the number of correctly predicted samples over the total number of samples.

## Results

### Group comparisons

**Fig 4:**
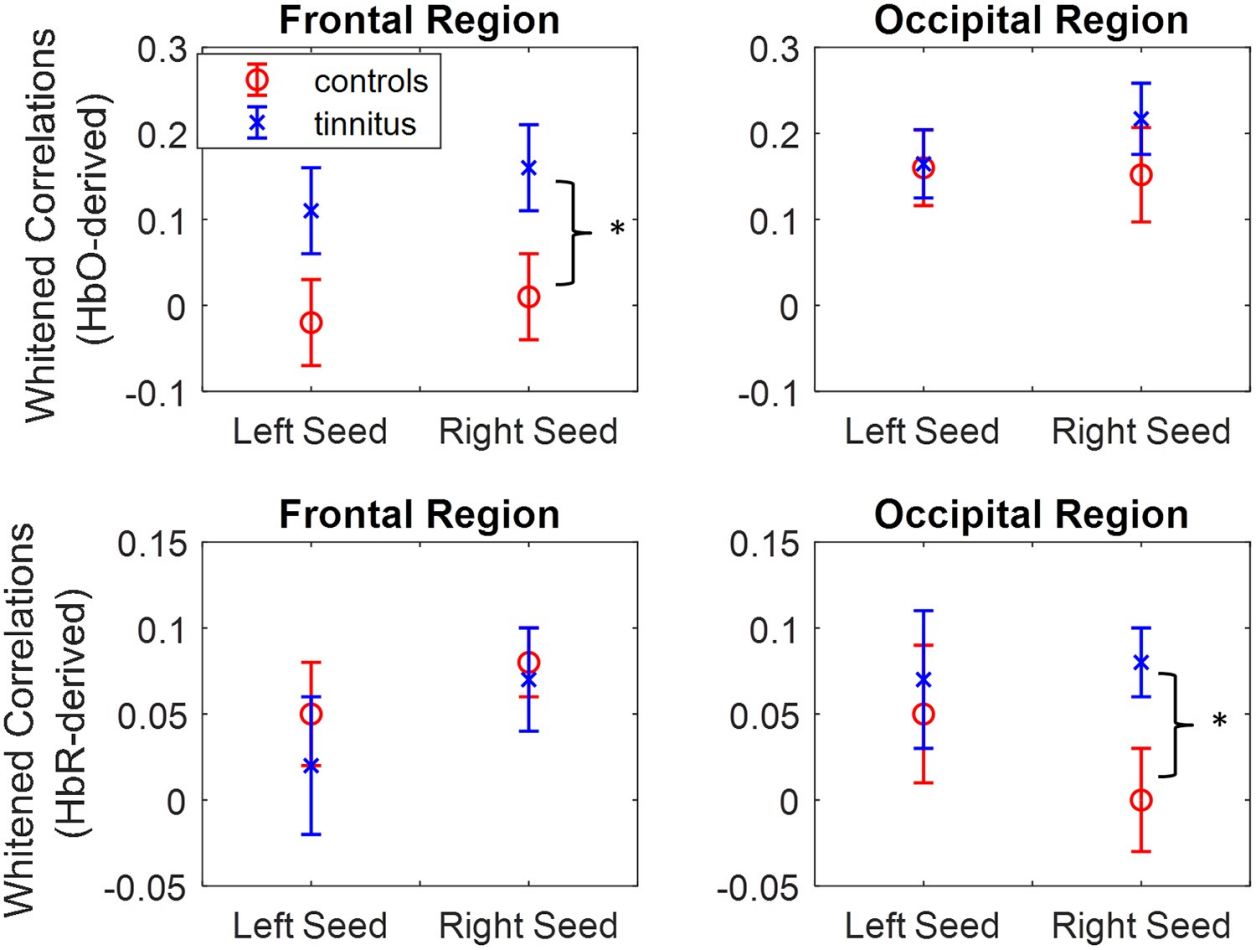
Hbo and HbR-derived whitened Correlations (measure of connectivity) between left and right seeds with frontal and occipital channels. Mean and SEM of correlations between seeds and channels in each region are shown. * *p* < 0.05.

*Resting state*: Differences between measures of connectivity between temporal seeds with frontal and occipital ROIs are shown in Fig 4. More detailed connectivity patterns with individual channels in frontal and occipital channels are shown in S1 and S2 Figs. Connectivity measures between both left and right seeds with frontal HbO signals were higher in the tinnitus group, with right seed differences reaching significance (right seed: *t*(41) = −2.125, *p* = 0.040; left seed: *t*(41) = −1.856, *p* = 0.071; *uncorrected p*). Right seed- occipital connectivity values derived from HbR signals were significantly higher in the tinnitus group *t*(41) = −2.266, *p* = 0.029; *uncorrected p*). This was not found for left seed connectivity.

#### Evoked responses

**Fig 5:**
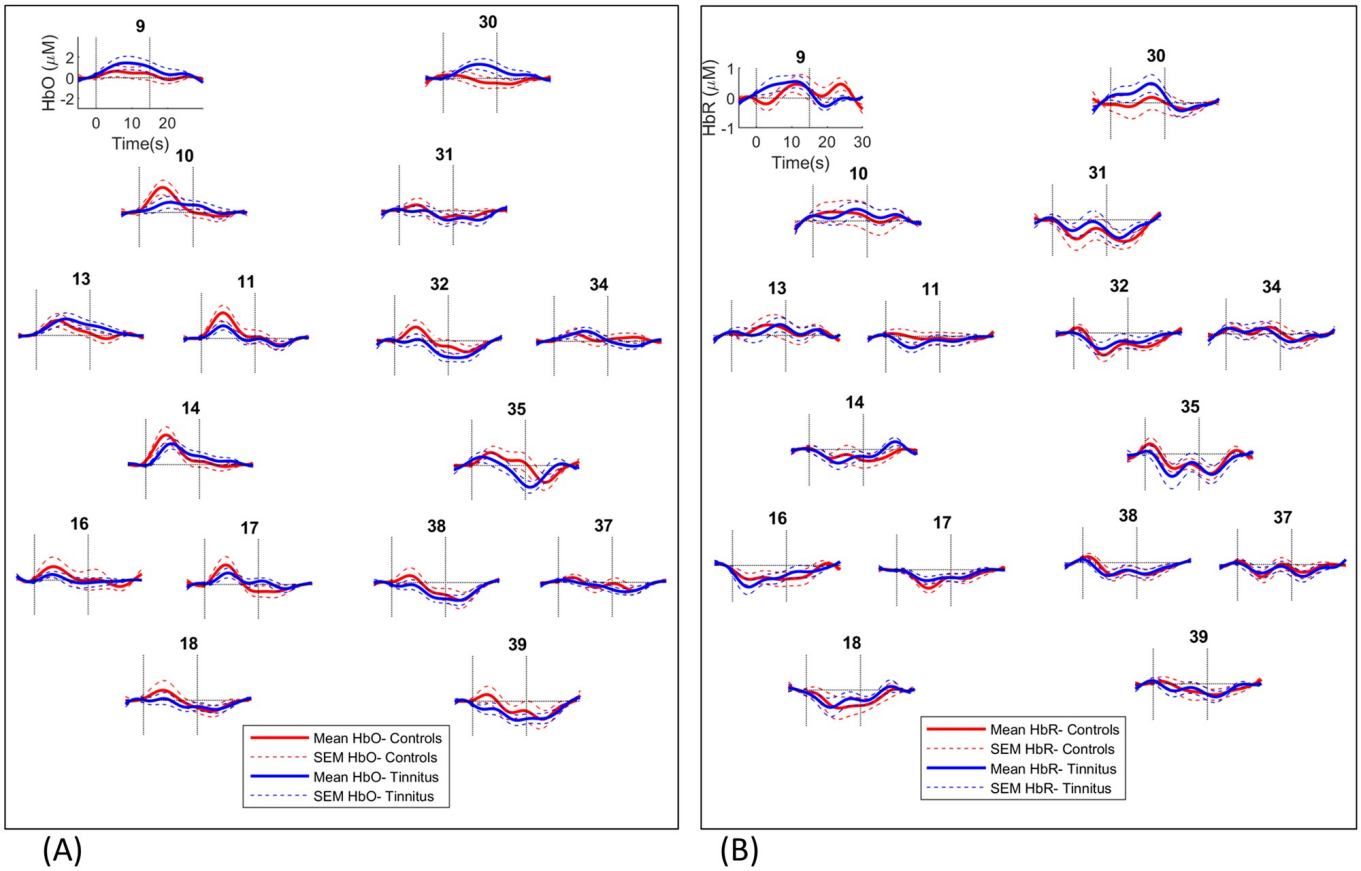
Group averaged auditory responses recorded from channels over the left and right temporal cortex. HbO (A) and HbR (B) responses shown. Channel numbers are shown above plots. Vertical lines show stimulus onset and offset times at 0 and 15 seconds.

**Fig 6:**
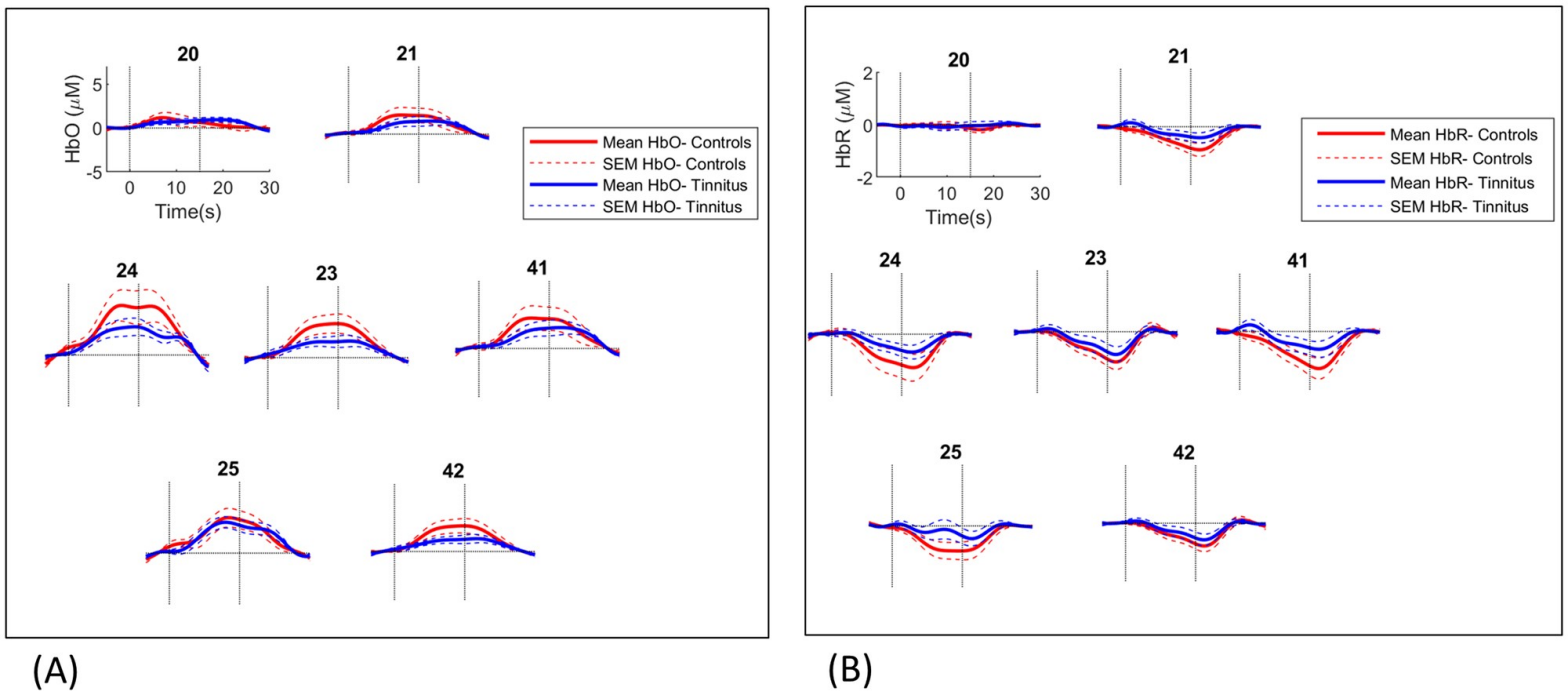
Group averaged visual responses recorded from occipital channels over the cuneus and superior occipital gyrus. HbO (A) and HbR (B) responses shown. Channel numbers are shown above plots. Vertical lines show stimulus onset and offset times at 0 and 15 seconds.

Group averaged auditory and visual evoked responses are shown in Figs 5 and 6.

Auditory response amplitudes averaged over the first five seconds following stimulus onset were compared between left and right auditory regions using paired t-tests and between groups using independent sample t-tests. This period was chosen to capture rise time or onset of the response which has been shown in previous work to last on average 5–6 seconds [38, 39]. Our previous research on fNIRS auditory evoked responses, identified a transient response showing a clear peak within the stimulation period [18]. There was no significant difference between left and right auditory responses. Averaged across both sides, the auditory response was smaller in the tinnitus group (*t*(39) = −2.199, *p* = 0.034; *uncorrected p*). This group difference was not found for HbR responses.

As seen in Fig 6, the visual response was more sustained in duration following stimulus onset compared to the auditory response. This is in agreement with previous fNIRS research on activation of the occipital cortex to checkerboard stimuli which showed a rise in HbO and fall in HbR concentration for around 10 seconds after stimulus onset as well as a slow return to baseline following stimulus offset which lasted around 10 seconds [19].

A t-test showed response amplitudes averaged over 10–15 seconds following stimulus onset were significantly larger in the control group (t(41) = 2.301, p 0.027; uncorrected *p*).

**Fig 7:**
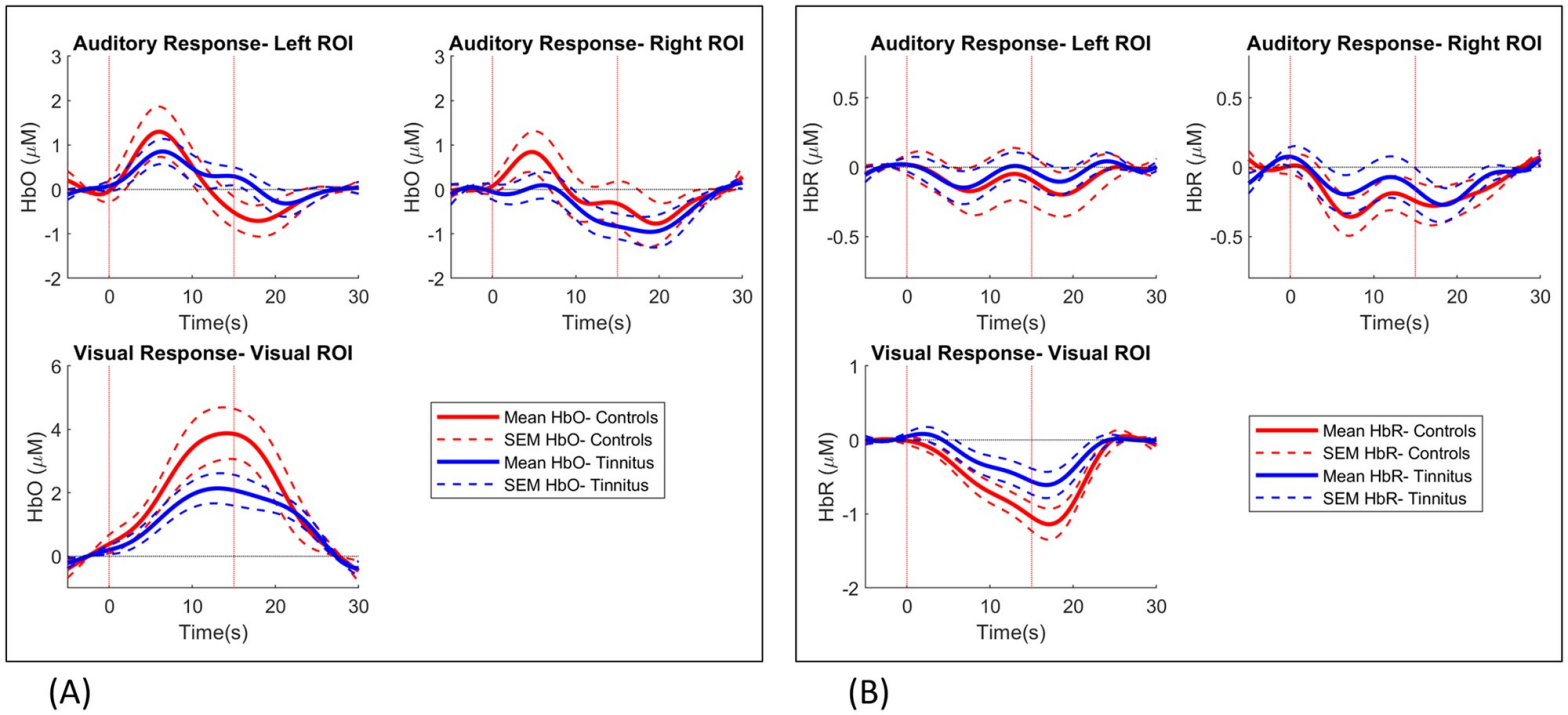
Group averaged auditory and visual responses. HbO (A) and HbR (B) responses averaged over auditory and visual ROIs. ROI: region of interest.

Fig 7 shows responses averaged over channels in left and right temporal regions and the occipital region. Since statistical analysis was performed with response amplitudes averaged over regions, this figure allows visual inspection of the waveforms averaged over the auditory and visual channels. Auditory responses showed a clear onset or rise lasting around 5 seconds after stimulation. Visual responses were more sustained with a slower rise lasting around 15 seconds after stimulation.

### Change in fNIRS measures with demographics and tinnitus severity

**Fig 8:**
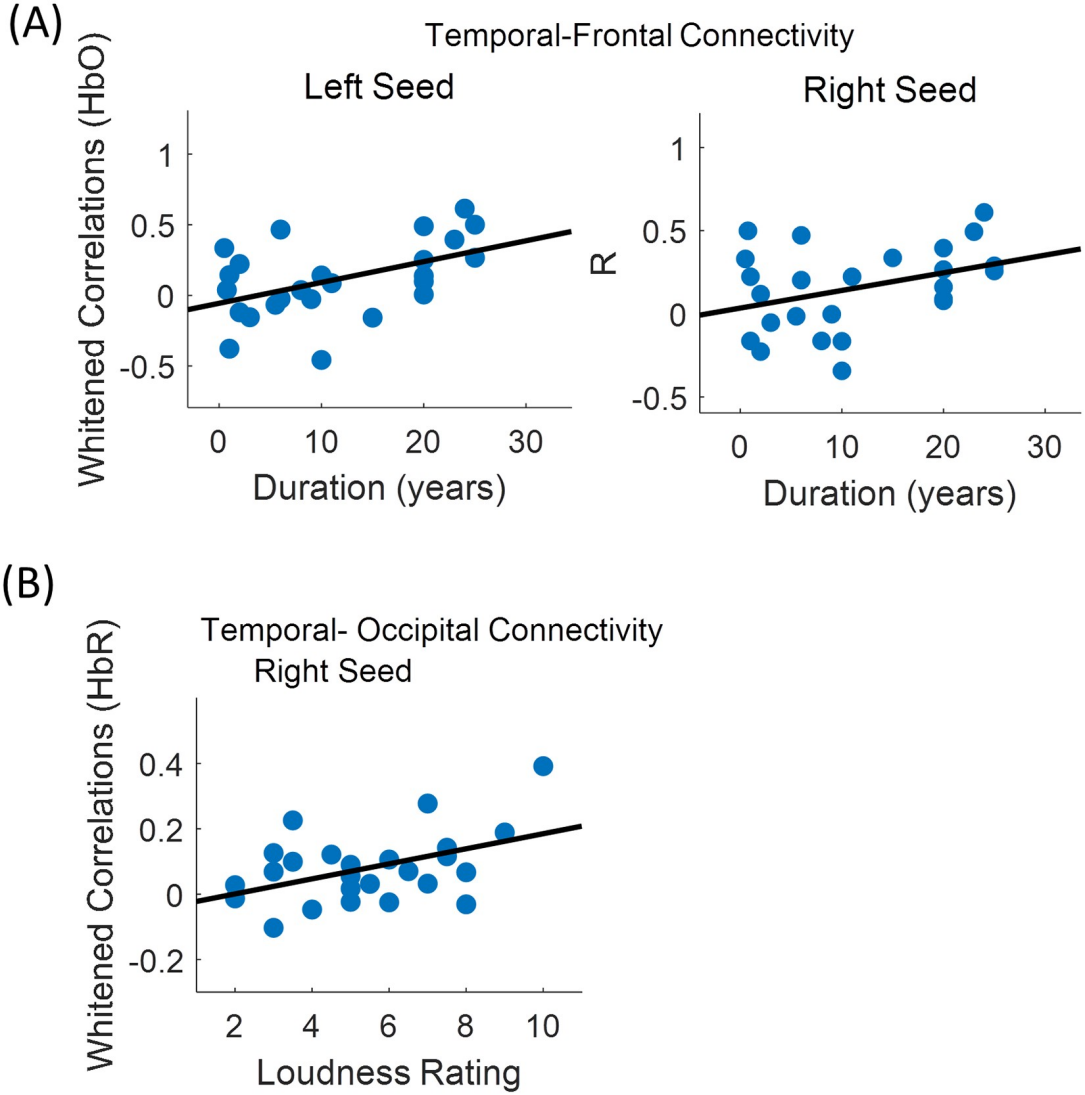
Change in connectivity with duration of tinnitus and loudness. (A) Change in HbO derived temporal- frontal connectivity with duration of tinnitus. (B) Change in HbR derived temporal- occipital connectivity with subjective ratings of loudness.

Changes in fNIRS measures with tinnitus severity as assessed by the THI score, age, duration of tinnitus, hearing thresholds at 4 and 8 KHz and subjective ratings of loudness and annoyance were assessed using multiple linear regression. HbO-derived connectivity between left and right seeds and frontal channels increased with duration of tinnitus (Fig 8A) with the correlation on the right side approaching significance (*β* = 0.021, *SE* = 0.011, *p* = 0.078). HbR-derived connectivity between the right seed and occipital channels (Fig 8B) increased significantly with subjective ratings of loudness (*β* = 0.036, *SE* = 0.012, *p* = 0.01, *uncorrected p*). All other comparisons were non-significant.

### Feature combination using machine learning

Individual channel, rather than ROI averaged, auditory, visual and resting state fNIRS feature sets, either alone or in combination, were used with classifiers. Features were weighted (or ranked) by applying the feature extraction method, Information Gain. The best accuracy using only a single feature set was able to separate tinnitus participants from controls using auditory alone features weighted above 0.45 and a Naïve Bayes classifier, resulting in an accuracy of 78.3% (Table 3). The weighting criterion resulted in 36 auditory features being used (20 HbO and 16 HbR derived auditory response amplitudes). Combining auditory, visual and resting state features weighted above 0.56 and using Rule Induction, Naïve Bayes and Neural Networks classifiers also resulted in accuracies above 70%. Features used included 19 auditory, 17 visual and 22 resting state connectivity measures. Of these total 58 features, 35 were derived from HbO and 23 from HbR signals. Connectivity measures in the selected features contained more right-seed features than left and more temporal-occipital features compared to temporal-frontal ones. The highest accuracy for classifying tinnitus participants from controls was achieved using Naïve Bayes classifier with auditory features (Table 3). The highest sensitivity was also achieved using Naïve Bayes with features from all three conditions selected using Information Gain. The Artificial Neural Network algorithm resulted in similar sensitivity and specificity values of 71.41% and 74.62% respectively. KNN was also used to classify tinnitus participants from controls however resulted in a low accuracy (~60%).

**Table 3:** Classifiers and features with highest accuracy for predicting participants with tinnitus and controls.

Classifier	features	Sensitivity	Specificity	Accuracy
Naïve Bayes	Auditory response	72.33%	64.25%	78.3%
Rule Induction	Combined auditory, visual and connectivity	80.66%	67.33%	75.09%
Naïve Bayes	Combined auditory, visual and connectivity	86.42%	61.25%	74.75%
Neural Network	Combined auditory, visual and connectivity	71.41%	74.62%	72.33%

Table 4 shows classification results for differentiating slight/ mild (n = 18) from moderate/ severe (n = 7) tinnitus. To categorise these tinnitus participants, the highest accuracies (above 75%) were achieved using connectivity measures weighted above 0.45, with Neural Network, KNN and Rule Induction classifiers (Table 4). A total of 48 features (23 HbO and 25 HbR derived auditory response amplitudes) were included with most features from right-seed HbR temporal- frontal and temporal occipital measures. Highest sensitivity (correctly predicting those with moderate/ severe tinnitus) and accuracy was achieved using the Neural Network classifier although low specificity of 51.23% was obtained.

**Table 4:** Classifiers and features with highest accuracy for predicting severity of tinnitus (slight/ mild n = 18, versus moderate/ severe n = 7) as rated using the Tinnitus Handicap Inventory (THI).

Classifier	features	Sensitivity	Specificity	Accuracy
Neural network	Connectivity features	51.23%	95.12%	87.32%
KNN(K = 1)	Connectivity features	50.86%	90.21%	81.22%
Rule Induction	Connectivity features	34.63%	90.06%	76.53%

## Discussion

Results from this study demonstrate that fNIRS can be used to differentiate patients suffering from tinnitus from controls and have identified fNIRS features that are associated with subjective ratings of tinnitus severity. In comparison to the control group, higher temporal-frontal HbO connectivity as well as higher right seed temporal-occipital HbR connectivity was found in the tinnitus group. The former measure showed an increase with duration of tinnitus while the latter increased significantly with subjective ratings of loudness. Auditory and visual responses were found to be reduced in the tinnitus group compared to controls but did not change significantly with tinnitus severity. In agreement with this, the machine learning algorithms have classified individuals with tinnitus and controls using features from resting state and evoked responses while the highest classification accuracy for patients with tinnitus at different severity levels was achieved using only connectivity features.

### Resting state temporal-frontal connectivity

A number of recent neuroimaging studies investigated altered resting state brain connectivity in patients with tinnitus [1, 4, 9]. Our study is in agreement with most of these studies, in demonstrating an increased connectivity among auditory and non-auditory brain regions such as the frontal and dorsolateral prefrontal cortex [1]. Connectivity among auditory and non-auditory brain regions such as the prefrontal region is known to play an important role in conscious sound perception including tinnitus [1, 40, 41]. Some studies have suggested the increased connectivity to be associated with the distress experienced by tinnitus patients [1]. Our findings have shown an increasing trend between temporal-frontal connectivity and duration of tinnitus (Fig 8A). This difference may be due to differences in how distress is measured (here we have only asked about annoyance). Duration has been identified as an important factor in tinnitus studies as it has possible effects on plasticity and habituation [15]. A study by Schmidt et al. comparing patients with tinnitus with a control group, showed disruption to the default mode network (a resting state network in the brain) in the tinnitus group that appeared to occur over time in patients [42]. Although this measure would need to be explored further to highlight what aspect of tinnitus it is measuring, an important finding is its independence of the tinnitus loudness experienced by patients suggesting the two can be measured separately.

### Resting state temporal-occipital connectivity and visual responses

In our study, right seed temporal-occipital connectivity was found to be increased in patients with tinnitus. The fNIRS channels in the occipital region covered the cuneus and superior occipital gyrus. The cuneus is located in the occipital part of the brain and is involved in visual processing. It is proposed that due to the existence of neural pathways between auditory and visual regions, tinnitus-related abnormal activity in the auditory cortex can lead to altered activity in the cuneus [9, 16]. Activation of the auditory cortex and integration of multi-sensory audio-visual information has been suggested to affect the perceived loudness of tinnitus [1, 7]. This is in agreement with our findings showing temporal-occipital connectivity to be associated with subjective ratings of loudness but not annoyance (Fig 8B). As mentioned earlier, this suggests that loudness and annoyance can be measured independently using fNIRS. This is important as studies on the delivery of tinnitus services have shown it is the perception of loudness that patients specifically want reduced [3]. Altered activity in the cuneus may also lead to the reduced visual responses observed in our study (Fig 7).

The increase in connectivity of the right temporal seed with frontal and occipital channels, but not left, is of interest as asymmetry in brain activity has been reported as a feature in many tinnitus imaging studies [1, 2, 14]. Comparison of resting state activity in participants with tinnitus and those without using positron emission tomography (PET), has shown stronger asymmetry in auditory cortex activity in participants with tinnitus compared to controls [43, 44]. Asymmetry in brain activity in different frequency bands has also been shown in tinnitus electroencephalography (EEG) and magnetoencephalography (MEG) resting state studies [10, 45]. A number of functional magnetic resonance imaging (fMRI) studies measuring sound evoked activity in participants with tinnitus and those without have shown lateralised responses in the auditory cortex in participants with tinnitus [9]. It remains inconclusive whether asymmetry in activity is dependent on which side tinnitus is perceived or whether it is perceived unilaterally or bilaterally [2].

### Sound evoked auditory responses

Contrasting findings have been reported from studies measuring sound-evoked activity in patients with tinnitus and controls using fMRI [2]. Melcher et al. showed smaller responses in the inferior colliculus of patients with tinnitus whereas other studies showed increased responses [46, 47]. Although scanner noise has been suggested as contributing to the smaller evoked response, another explanation has been the increased background neural activity present in tinnitus leading to saturation of the hemodynamic response [48]. Since fNIRS involves no background noise, the latter could explain the reduced auditory responses in patients with tinnitus compared to controls. The negative variation seen in Fig 7 (right auditory ROI) could be explained by reduced neural activity. Negative hemodynamic responses are attributed to two main mechanisms [49]. The first is a reduction or suppression of neural activity and the second, a ‘blood stealing’ effect [50, 51]. The latter effect proposes that an increase in blood flow in an activated cortical region causes a decrease in blood flow in an adjacent region.

### Using machine learning with fNIRS features

When comparing tinnitus participants with controls, three classifiers Naïve Bayes, Rule Induction and Neural Networks showed the highest accuracies (Table 3). Naive Bayes showed the highest accuracy with only auditory response features however specificity with this method was only ~64%. Also, auditory features alone did not result in high accuracy when used to predict severity level within the tinnitus group (Table 4). The Neural Network classifier resulted in similar sensitivity and specificity (~71% and 74% respectively) when using features from all three recording conditions. This classifier also resulted in high specificity when used to predict severity of tinnitus within the tinnitus group (Table 4). While Neural Network and Rule Induction both resulted in accuracies above 70% to classify tinnitus from controls and tinnitus with different severity levels, Neural Network has higher time complexity (i.e. time needed to run the algorithm) compared to Rule Induction. Rule Induction can also produce understandable rules which may help interpret findings. Both these classifiers will need to be explored with a larger dataset of tinnitus patients with more heterogeneity in terms of severity in order to determine their clinical applicability and to improve the sensitivity of the classifiers to separate tinnitus severity levels as currently they only reach ~50% (Table 4).

When classifying tinnitus severity, the best performance was achieved when connectivity features from resting state recordings alone were used (Table 4). This could be due to the stimuli, in particular the auditory stimulus, somewhat masking the tinnitus during recordings. While evoked responses appear to contribute to the accuracy of detecting the *presence* of tinnitus, resting state recordings alone seem appropriate for grading tinnitus severity.

Both HbO and HbR features were used with feature extraction and classification. Based on the results a similar number of each feature type contributed to the classification and therefore using both HbO and HbR features appears to result in better performance compared to HbO alone. This is despite most studies reporting only HbO findings as the signals have higher signal to noise ratio compared to HbR [52], which is consistent with our statistical analysis for which HbO identified more group differences than HbR.

A reduction in the number of channels would assist in developing a more clinically usable fNIRS cap with fewer sources and detectors. In this study, we have used features from all channels (Fig 2) allowing the feature extraction algorithm to select the most relevant, which for evoked responses have been mostly from the relevant anatomical regions (e.g. auditory response features from auditory channels). With further data, feature extraction can eliminate redundant channels and features, leading to faster computation time.

The machine learning algorithms were run on an Intel Core i7 7600U system with 2.8GHz CPU and 16 GB of RAM. The total time to run the algorithms included model training time plus inference time. Inference time refers to the time taken to predict the classification of a test sample. The inference time for all algorithms was negligible at less than 1 second. For Neural Networks, the total processing time was 150 seconds while for the other algorithms presented in Tables 3 and 4, it was less than 3 seconds. For clinical applications, the time taken to train a model is not a limiting factor as this phase is done offline.

Our findings so far show feasibility of a machine learning model trained to classify an individual’s fNIRS data to a tinnitus severity level. Repeating recordings after a certain treatment and running these through the same model would be able to highlight changes in severity as a result of intervention.

### Limitations and future work

A limitation of the imaging technology used in this study is the limited cortical depth that near-infrared light can penetrate; meaning changes in HbO and HbR levels in deep cortical regions cannot be measured. In our study, we have focused on cortical regions accessible using fNIRS that have been associated with tinnitus using other imaging techniques such as fMRI and PET. However, there may be limitations in terms of subtypes of tinnitus that we are able to measure using this technique.

An important aspect in tinnitus studies is the choice of the control group [1]. While we have matched the two groups in age and hearing loss there may be other factors such as depressive symptoms and hyperacousis which would need to be considered in future studies.

In the machine learning algorithms used in this study we have used fNIRS evoked response amplitudes as well as measures of connectivity from resting state data. Evoked responses may be better quantified using different features characterising temporal and spectral content of the waveforms [26]. In regards to connectivity measures, our current analysis of resting state data has used seed analysis as a time-domain method. Other methods both in time and frequency domains are also used to investigate resting state connectivity and may provide further insight into our data [32]. Using more advanced methods of feature extraction to improve characterising time-series data could increase accuracy of our classification algorithms.

As with many objective clinical measures, in this study we are using subjective ratings of tinnitus to develop an objective measure. Tinnitus by nature will always have a subjective component however an objective measure will help measure certain aspects of tinnitus that will assist with development and trial of new treatments.

## Conclusion

Our statistical findings support previous research which has identified measures of brain connectivity or evoked responses associated with tinnitus. We have built on these findings further using fNIRS and machine learning and have identified visual and auditory evoked response and resting state connectivity features which differentiate tinnitus patients from controls and temporal-occipital connectivity features which classify tinnitus patients to a low versus high severity level. Using fNIRS has a number of advantages over other imaging techniques such as portability and lack of scanner noise which make it suited to eventual clinical use. This approach will greatly assist in addressing a critical unmet need of developing an objective measure of tinnitus that can be used clinically. Findings from this study will have further applications in identifying subtypes of tinnitus, objectively assessing the effectiveness of tinnitus treatments and a better understanding of brain networks involved in this condition.

## Supporting information

S1 Fig Group averaged whitened correlation maps. HbO-derived whitened correlations between left and right seeds with individual frontal and occipital channels.
(TIF)

S2 Fig Group averaged whitened correlation maps. HbR-derived whitened correlations between left and right seeds with individual frontal and occipital channels.
(TIF)

S1 Dataset Resting state and evoked response features extracted from fNIRS signals. (XLSX)
