# Correction: Objective measurement of tinnitus using functional near-infrared spectroscopy and machine learning

**DOI:** 10.1371/journal.pone.0359852

**Published:** 2026-10-01

**Authors:** Mehrnaz Shoushtarian, Roohallah Alizadehsani, Abbas Khosravi, Nicola Acevedo, Colette M. McKay, Saeid Nahavandi, James B. Fallon

In Fig 1, the y-axis label is incorrectly listed as “Sound Intensity (dB SPL)”. The correct label is “Hearing Level (dB HL)”. Please see the correct Fig 1 here.

**Fig 1:**
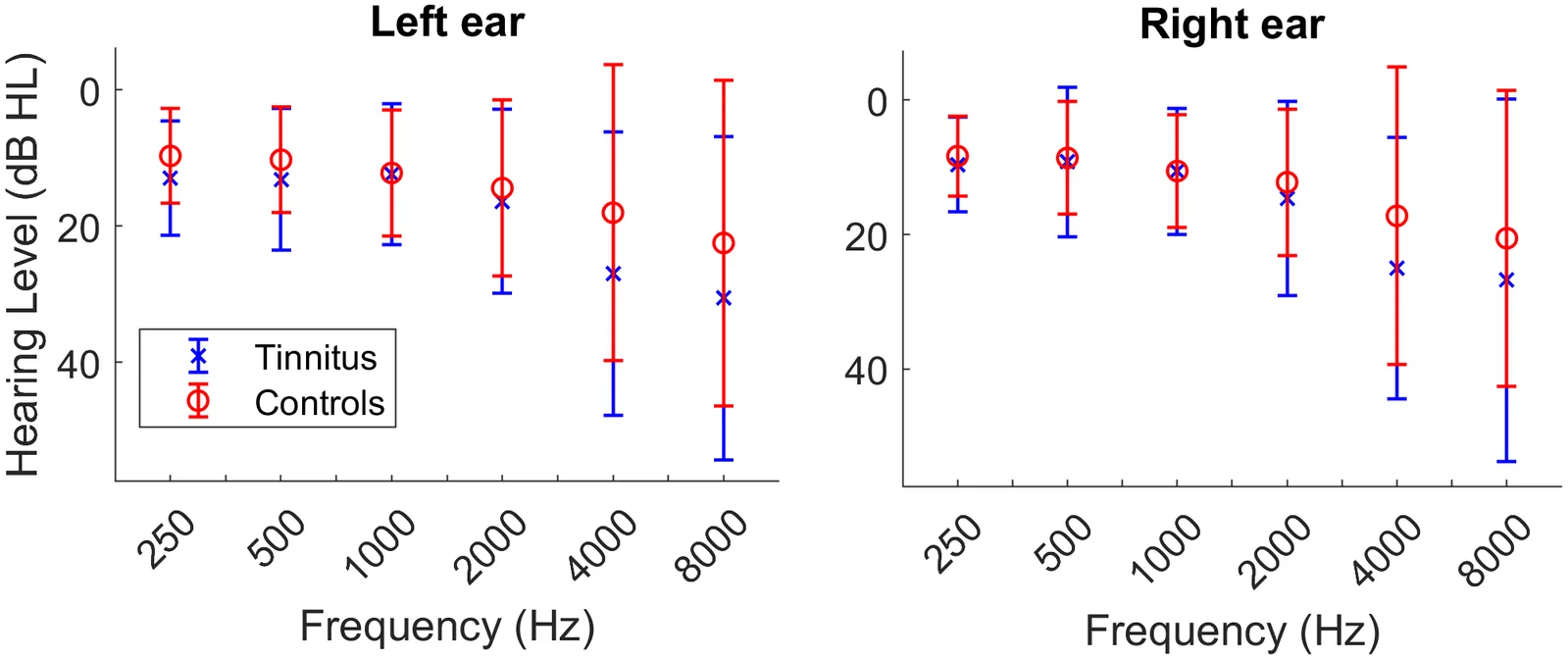
Mean (SD) of hearing thresholds in participants with tinnitus and controls.
