# Supplementary figures and images for: Objective measurement of tinnitus using functional near-infrared spectroscopy and machine learning
Source: PLoS One. 2020 Nov 18;15(11):e0241695. doi: 10.1371/journal.pone.0241695 (PMC7673524; doi:10.1371/journal.pone.0241695)

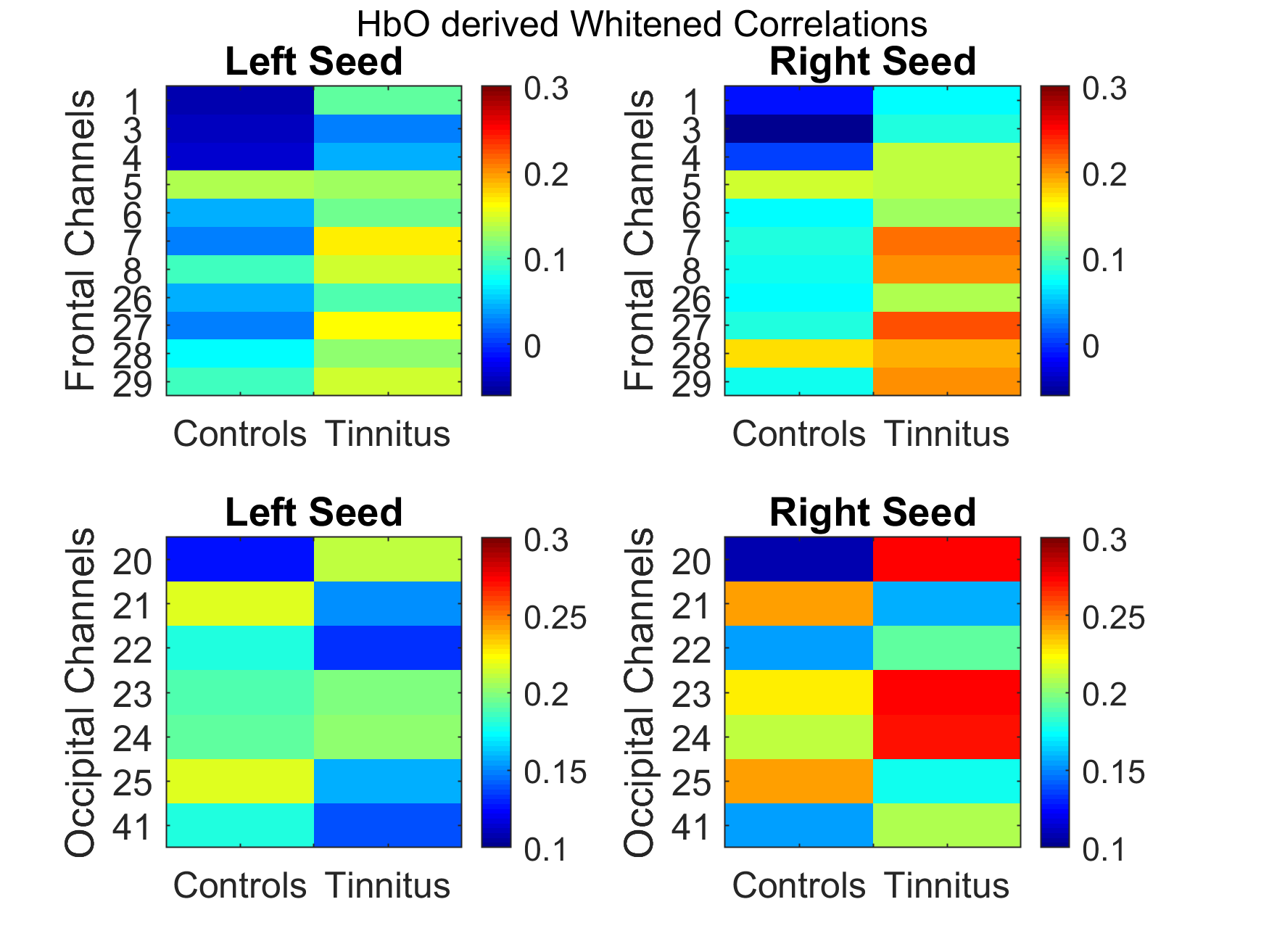

Supplement: S1 Fig — HbO-derived whitened correlations between left and right seeds with individual frontal and occipital channels. (TIF) [file pone.0241695.s001.tif]

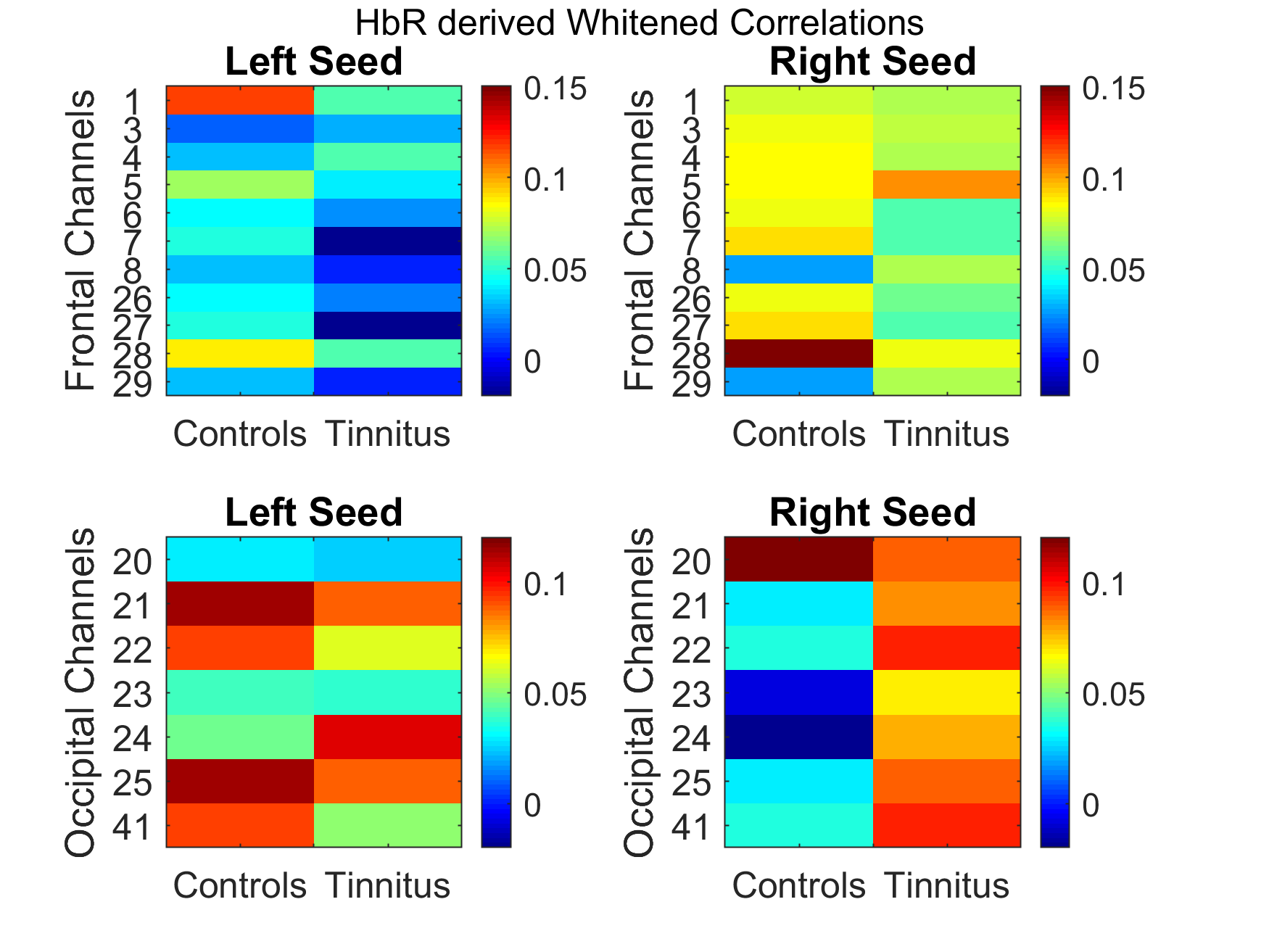

Supplement: S2 Fig — HbR-derived whitened correlations between left and right seeds with individual frontal and occipital channels. (TIF) [file pone.0241695.s002.tif]
